# The evolution of infectious transmission promotes the persistence of *mcr-1* plasmids

**DOI:** 10.1128/mbio.00442-23

**Published:** 2023-06-14

**Authors:** Jun Yang, Renjie Wu, Qiang Xia, Jingjing Yu, Ling-Xian Yi, Ying Huang, Meixin Deng, Wan-Yun He, Yuman Bai, Luchao Lv, Vincent Burrus, Chengzhen Wang, Jian-Hua Liu

**Affiliations:** 1 College of Veterinary Medicine National Risk Assessment Laboratory for Antimicrobial Resistant of Microorganisms in Animals, Guangdong Provincial Key Laboratory of Veterinary Pharmaceutics Development and Safety Evaluation, Key Laboratory of Zoonosis of Ministry of Agricultural and Rural Affairs South China Agricultural University, Guangzhou, China; 2 Guangdong Laboratory for Lingnan Modern Agriculture, Guangzhou, China; 3 College of Mathematics and Informatics, South China Agricultural University, Guangzhou, China; 4 Département de biologie, Université de Sherbrooke, Sherbrooke, Québec, Canada; Vanderbilt University, Nashville, Tennessee, USA; University of Idaho, Moscow, Idaho, USA

**Keywords:** *mcr-1*, plasmid stability, evolution, conjugation

## Abstract

Conjugative plasmids play a vital role in bacterial evolution and promote the spread of antibiotic resistance. They usually cause fitness costs that diminish the growth rates of the host bacteria. Compensatory mutations are known as an effective evolutionary solution to reduce the fitness cost and improve plasmid persistence. However, whether the plasmid transmission by conjugation is sufficient to improve plasmid persistence is debated since it is an inherently costly process. Here, we experimentally evolved an unstable and costly *mcr-1* plasmid pHNSHP24 under laboratory conditions and assessed the effects of plasmid cost and transmission on the plasmid maintenance by the plasmid population dynamics model and a plasmid invasion experiment designed to measure the plasmid’s ability to invade a plasmid-free bacterial population. The persistence of pHNSHP24 improved after 36 days evolution due to the plasmid-borne mutation A51G in the 5′UTR of gene *traJ*. This mutation largely increased the infectious transmission of the evolved plasmid, presumably by impairing the inhibitory effect of FinP on the expression of *traJ*. We showed that increased conjugation rate of the evolved plasmid could compensate for the plasmid loss. Furthermore, we determined that the evolved high transmissibility had little effect on the *mcr-1*-deficient ancestral plasmid, implying that high conjugation transfer is vital for maintaining the *mcr-1*-bearing plasmid. Altogether, our findings emphasized that, besides compensatory evolution that reduces fitness costs, the evolution of infectious transmission can improve the persistence of antibiotic-resistant plasmids, indicating that inhibition of the conjugation process could be useful to combat the spread of antibiotic-resistant plasmids.

**IMPORTANCE:**

Conjugative plasmids play a key role in the spread of antibiotic resistance, and they are well-adapted to the host bacteria. However, the evolutionary adaptation of plasmid-bacteria associations is not well understood. In this study, we experimentally evolved an unstable colistin resistance (*mcr-1*) plasmid under laboratory conditions and found that increased conjugation rate was crucial for the persistence of this plasmid. Interestingly, the evolved conjugation was caused by a single-base mutation, which could rescue the unstable plasmid from extinction in bacterial populations. Our findings imply that inhibition of the conjugation process could be necessary for combating the persistence of antibiotic-resistance plasmids.

## INTRODUCTION

Horizontal gene transfer (HGT) improves genetic diversity by transferring genes between different species. Bacterial conjugation is an important mechanism of HGT (1). It transfers genetic material from donor bacteria to recipient bacteria in direct contact and helps bacteria adapt to a new environment, playing a significant role in the evolution of bacteria (2, 3). The transmission of antibiotic resistance genes (ARGs) between bacteria via plasmids is strong evidence of bacterial adaptation, becoming a considerable threat to human health worldwide in recent decades (4 - 6). As the horizontal transfer (HT) of resistance genes between microorganisms via plasmids is highly efficient, conjugative plasmids are considered the main drivers of antimicrobial resistance spread among clinically relevant pathogens (3). Thus, the emergence of plasmid-mediated resistance genes, especially those conferring resistance to last-line antimicrobials such as colistin, has caused global concern. The *mcr-1* gene confers resistance to colistin by modifying the lipid A of lipopolysaccharide (7 - 11). So far, *mcr-1* has spread globally and has recently been classified as the highest-risk ARG in an omics-based framework for assessing the health risk of ARGs based on gene mobility, human-associated-enrichment, and host pathogenicity (12). Some resistance plasmids show high persistence even without antibiotic selection, especially F-like plasmids from Enterobacteriaceae (13 - 16). The persistence of plasmids in bacterial populations is usually influenced by plasmid stability function (including efficient replication, partition system, and post-segregational killing system), plasmid cost, and conjugational transfer.

Poor plasmid persistence in bacterial populations can be improved by compensatory evolution occurring in the bacterial host, plasmid, or both by reducing the fitness burden (17 - 22). Several studies have shown that mutations in host-encoded genes can often reduce plasmid cost and improve persistence (18, 19, 21, 22). For example, chromosomal compensatory mutations in *Pseudomonas fluorescens* that target the two-component system GacA/GacS or the hypothetical protein PFLU4242 reduce the fitness cost of pQBR plasmids and increase their permissiveness (18, 22). Several studies have shown that plasmid cost and persistence can often be improved by mutations in host-encoded helicases (19, 21). These chromosomal mutations compensated for the cost of plasmid carriage by reducing the metabolism burden caused by plasmid replication or transcription of the plasmid-encoded genes. Besides, a poor plasmid-host association can improve due to the evolution of plasmids (17, 20). Plasmid persistence can improve after losing costly conjugative transfer genes during long-term experimental evolution (20). In *Shewanella oneidensis* MR-1, loss of the helicase binding domain (DnaB) in the replication initiation protein encoded by an IncP-1β plasmid reduced the plasmid cost and improved long-term plasmid survival (17).

In addition to the selective benefit of plasmid-encoded genes and compensatory adaptation, plasmid conjugative transfer also influences its persistence. A previous study showed that a high transfer frequency could offset plasmid loss resulting from segregational loss and fitness cost (23, 24). We recently also found that the master conjugative regulator PixR promotes the persistence of *mcr-1*-positive IncX4 plasmids by increasing their transfer frequency (25). However, high plasmid transfer rates might impose a fitness burden on the host and slow its growth. In addition, high-throughput analysis of plasmid sequences showed that over half of known plasmids in nature lack a conjugation system (26 - 29). Hence, whether conjugation is the principal mechanism for plasmid persistence is debatable.

In this study, we evolved an unstable *mcr-1*-positive plasmid pHNSHP24 under colistin selection in *Escherichia coli* C600 for 36 days. Our results demonstrated that plasmid-encoded mutations that increase the plasmid transfer rate could help stabilize *mcr-1*-positive plasmids and rescue the costly *mcr-1* plasmid from extinction in the bacterial populations.

## RESULTS

**Fig 1:**
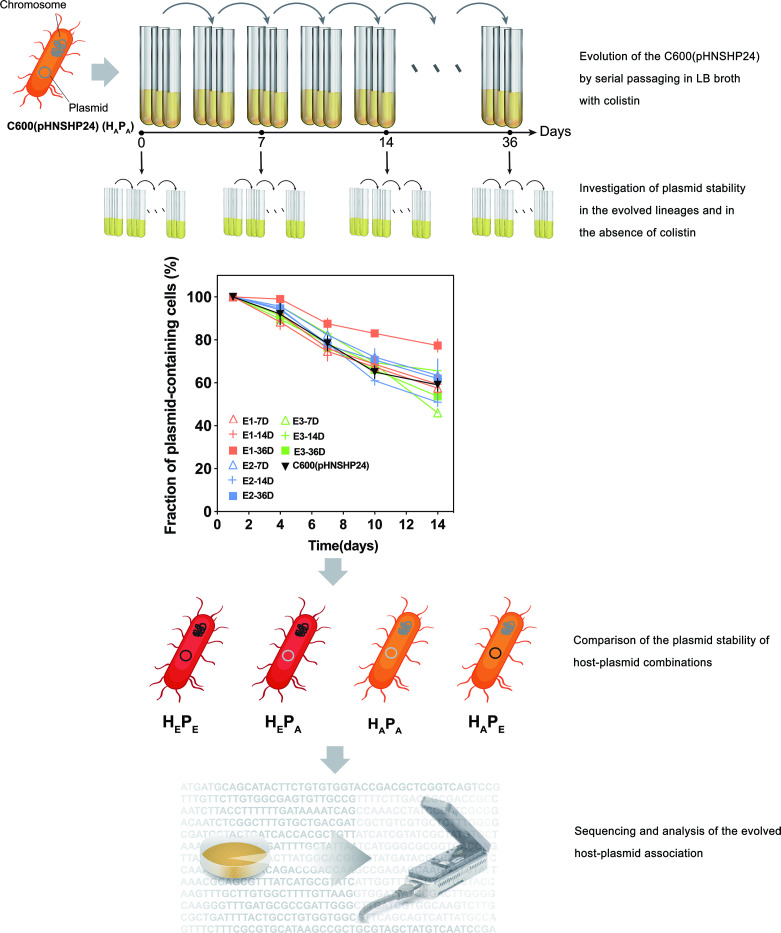
Experimental overview. Three lineages (E1–E3) of the *Escherichia coli* C600 containing pHNSHP24 plasmid were cultured by serial batch transfer in LB broth and in the presence of colistin. The plasmid persistence was done at day 0 as well as following 7, 14, and 36 days of serial transfer. The E1 lineage evolved for 7, 14, and 36 days was named C600(pHNSHP24-7D), C600(pHNSHP24-14D), and C600(pHNSHP24-36D), respectively. To determine if the improvement of plasmid persistence was due to evolution of the plasmid, the host, or both, the plasmid persistence was measured for all possible host-plasmid permutations: the ancestral host (H_A_) containing the ancestral plasmid (P_A_); the ancestral host (H_A_) containing the evolved plasmid (P_E_); the evolved host (H_E_) containing the ancestral plasmid (P_A_); the evolved host (H_E_) containing the evolved plasmid (P_E_). The genome sequences of the evolved host-plasmid associations were analyzed to determine the key mutations on plasmids or chromosomes.

*E. coli* C600 harboring pHNSHP24 was obtained by conjugation from *E. coli* SHP24 isolated from a swine rectal swab sample of the pig farm where we first discovered *mcr-1* in 2013 (30). pHNSHP24 is a hybrid between an IncFII plasmid and a phage-like pO111 plasmid carrying a copy of IS*Apl1*-flanked *mcr-1*. pHNSHP24 carries the whole conjugative transfer region of the IncFII plasmid. It contains a putative *stbAB* operon located in the IncFII plasmid part, known as a partitioning system, and two putative toxin-antitoxin systems, *hok*/*sok* and *doc*/*phd*, located in the phage-like pO111 part, which may mediate the stable maintenance of plasmid (30). The persistence of pHNSHP24 in *E. coli* C600 was investigated by serial passaging for 14 days without colistin. Since pHNSHP24 could not persist stably in *E. coli* C600 (40% plasmid-free cells after 14 days) (Fig. 1), *E. coli* C600(pHNSHP24) was used to investigate plasmid-host adaptation in the presence of colistin. Here, we chose the transconjugant C600(pHNSHP24) to perform a laboratory evolution experiment instead of the native strain because *E. coli* SHP24 contains four other plasmids besides the pHNSHP24 (30).

### Experimental evolution of pHNSHP24 during antibiotic selection in *E. coli*

To investigate whether plasmid-host adaptation could enhance the persistence of pHNSHP24, we performed a plasmid evolution experiment in liquid medium containing colistin (Fig. 1). *E. coli* C600 carrying pHNSHP24 was cultured consecutively for 36 days. We monitored plasmid persistence following 7, 14, and 36 days of serial transfer in Luria-Bertani (LB) broth containing colistin. As shown in Fig. 1, the 36-day evolved lineage E1 (E1-36D) showed a pronounced gain in plasmid persistence compared to the non-evolved lineage. No improvement was observed after 7 and 14 days or with the two other evolved lineages.

**Fig 2:**
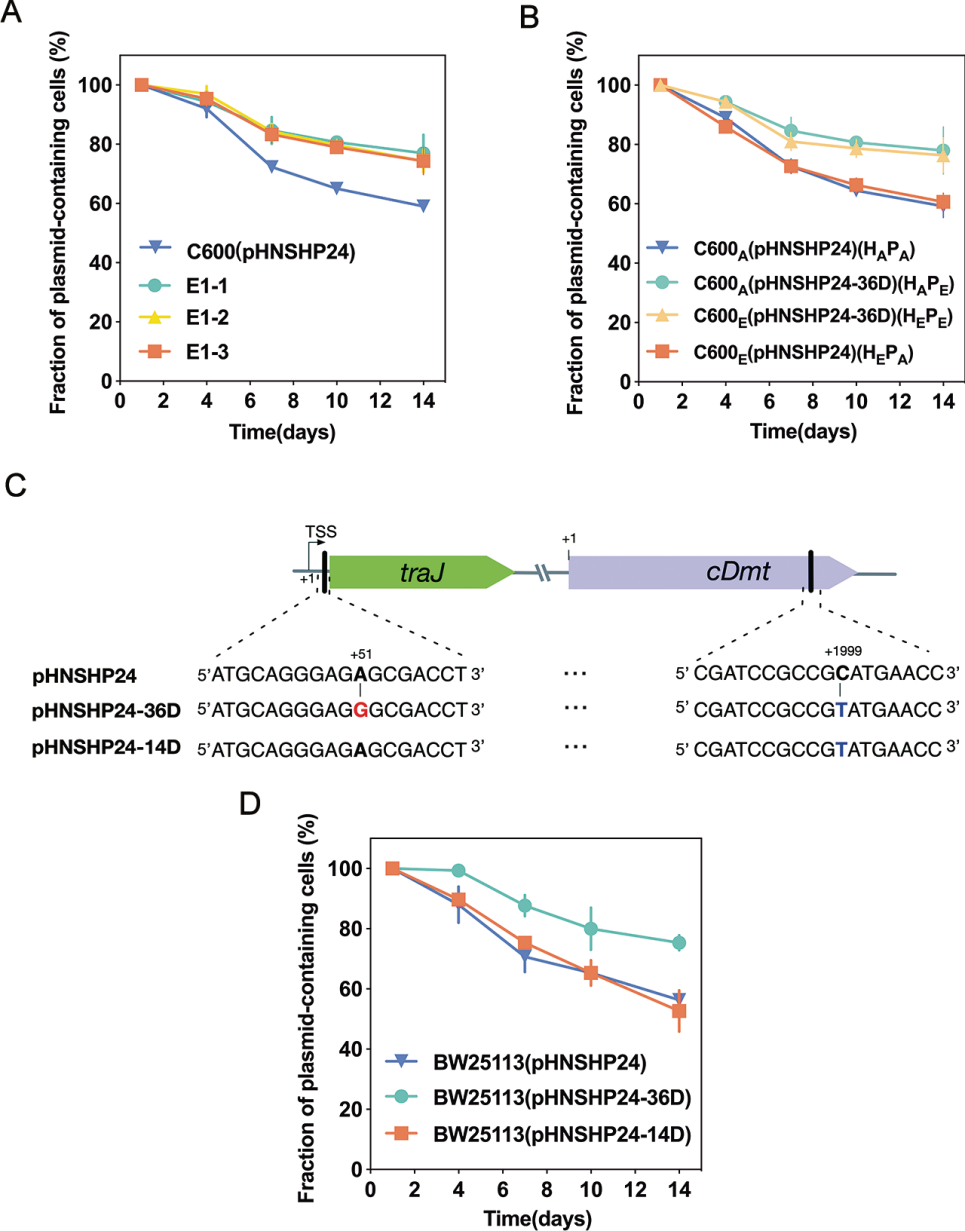
The A51G mutation in 5′UTR of *traJ* is responsible for the improved plasmid persistence. (**A**) The plasmid persistence data for the evolved clones (E1-1, E1-2, and E1-3). The E1-1, E1-2, and E1-3 clones were purified from the lineage E1 evolved for 36 days, and the persistence of plasmids from these clones was compared with the ancestral plasmid pHNSHP24 in *Escherichia coli* C600. (**B**) The plasmid persistence data for the ancestral host (H_A_) harboring the ancestral plasmid (P_A_) or the evolved plasmid (P_E_), and the evolved host (H_E_) harboring the ancestral plasmid (P_A_) or the evolved plasmid (P_E_). (**C**) The evolved plasmid-encoded mutations are located in the *cDmt* gene and the 5' untranslated region (UTR) of *traJ*. The evolved plasmids from E1-1, E1-2, and E1-3 clones were sequenced, and found that the sequences of each plasmid were identical. This evolved plasmid was named as pHNSHP24-36D. The pHNSHP24-36D plasmid contains C1999T mutation in *cDmt* gene and A51G in 5′UTR of *traJ*. The evolved plasmids from the lineage E1 evolved for 7 and 14 days were named as pHNSHP24-7D and pHNSHP24-14D, respectively. The sequences of pHNSHP24-7D and pHNSHP24-14D were identical, and they only contain C1999T mutation in *cDmt* gene (**D**) Comparison of the persistence of pHNSHP24, pHNSHP24-36D, and pHNSHP24-14D in *E. coli* BW25113. Each point represents the mean fraction of plasmid-containing cells (*n* = 3) and bars represent SD of the three biological replicates.

Then, we purified the lineage E1 evolved for 36 days by streaking on LB plates containing colistin and randomly picked three clones (E1-1, E1-2, and E1-3) to determine the plasmid persistence. As shown in Fig. 2A, all three strains exhibited improved plasmid persistence. To determine whether the enhanced plasmid persistence was due to the evolution of the plasmid or host, we transferred evolved pHNSHP24-36D (P_E_) from the E1-1 clone to the ancestral host *E. coli* C600_A_ (H_A_), and the resulting strain was named *E. coli* C600_A_(pHNSHP24-36D)(H_A_P_E_). The plasmid-free evolved strain E1-1 was obtained from the plasmid persistence experiment. Then, the ancestral plasmid pHNSHP24 (P_A_) was transferred into the plasmid-free strain E1-1 designated as *E. coli* C600_E_ (H_E_), and the resulting strain was named *E. coli* C600_E_(pHNSHP24)(H_E_P_A_). Then, we determined the plasmids (P_E_ and P_A_) persistence in H_A_ or H_E_. As shown in Fig. 2B, the evolved plasmid pHNSHP24-36D in both *E. coli* C600_A_ and *E. coli* C600_E_ shows dramatic improvement in persistence compared to the ancestral plasmid pHNSHP24. In contrast, we did not detect improved persistence of pHNSHP24 or pHNSHP24-36D in *E. coli* C600_E_ compared with *E. coli* C600_A_, indicating the evolution occurred in the plasmid, not the chromosome. We also compared the plasmid persistence profiles of H_A_P_A_, H_E_P_E_, H_A_P_E_, and H_E_P_A_, using the HT plasmid population dynamics model, as described in previous studies (21, 31). We used the Bayesian information criterion (BIC) to reflect the difference in plasmid persistence dynamics between each strain. As shown in Table 1, the plasmid persistence profiles of P_E_-bearing strains (H_A_P_E_, H_E_P_E_) showed a significant difference with P_A_-bearing strains (H_A_P_A_, H_E_P_A_) (the ΔBIC score was −38.6 to −30.17; the more negative the ΔBIC, the larger the difference between strains). However, the plasmid persistence profiles of host H_A_P_A_ or H_A_P_E_ were indistinguishable from that of the host H_E_P_A_ or H_E_P_E_, respectively (ΔBIC=19.17 or 19.47), further implying that the improved persistence was attributable to plasmid evolution.

**TABLE 1:** Statistical comparisons of persistence profiles for ancestral and evolved host-plasmid combinations, using Bayesian information criteria (BIC) (31)

Ancestral and evolved host-plasmid combinations^*b*^	BIC.sep−BIC.joint=ΔBIC^*a*^			
	H_E_P_A_	H_A_P_E_	H_A_P_A_	H_E_P_E_
H_A_P_A_	266.87–247.70=19.17	234.02–264.19=–30.17	–	234.57–268.82=–34.25
H_E_P_E_	242.37–280.97=–38.60	208.97–189.50=19.47	234.13–268.69=–34.56	–

^*a*^ BIC.sep−BIC.joint=ΔBIC was used to assess the magnitude of the difference between two plasmid persistence profiles (31). BIC.sep represents the BIC of the model which assumes the two plasmid persistence data are governed by different dynamics. BIC.joint represents the BIC of the “null” model which assumes that the stability dynamics of two data sets are the same. More negative ΔBIC values indicate larger differences between the two plasmid persistence data.

^*b*^ H_A_P_A_, the ancestral host harboring the ancestral plasmid; H_E_P_E_, the evolved host harboring the evolved plasmid; H_A_P_E_, the ancestral host harboring the evolved plasmid; H_E_P_A_, the evolved host harboring the ancestral plasmid.

### The evolved 5′UTR of *traJ* is responsible for plasmid persistence improvements

To examine the underlying mechanism responsible for improving plasmid persistence, we compared the plasmid sequences of pHNSHP24 and pHNSHP24-36D. As shown in Fig. 2C, we observed point mutations in *cDmt* (cytosine-specific DNA methyltransferase gene) and the upstream sequence (A51G) of *traJ* in pHSNHP24-36D. Besides, we also sequenced the plasmids pHNSHP24-7D and pHNSHP24-14D purified from the clones that evolved for 7 and 14 days in the E1 lineage and found that the two plasmids only contained the C1999T mutation in *cDmt* (Fig. 2C). To determine whether the mutations in *cDmt* and *traJ* were required for increased plasmid persistence, we further measured the plasmid persistence of pHNSHP24, pHNSHP24-36D, and pHNSHP24-14D in *E. coli* BW25113. As shown in Fig. 2D, pHNSHP24-36D showed better persistence in BW25113 than pHNSHP24 and pHNSHP24-14D, while pHNSHP24 and pHNSHP24-14D showed similar persistence. These findings indicated that the mutation in *cDmt* does not affect plasmid persistence as it was the only SNP between pHNSHP24 and pHNSHP24-14D. Likewise, the difference between the persistence ability of pHNSHP24-36D and pHNSHP24-14D suggests that the mutation A51G in the 5′UTR of *traJ* improves plasmid persistence but whether it is dependent on the presence of the *cDmt* mutation is unknown.

### A point mutation at 5′UTR of *traJ* increases the plasmid conjugation rate

**Fig 3:**
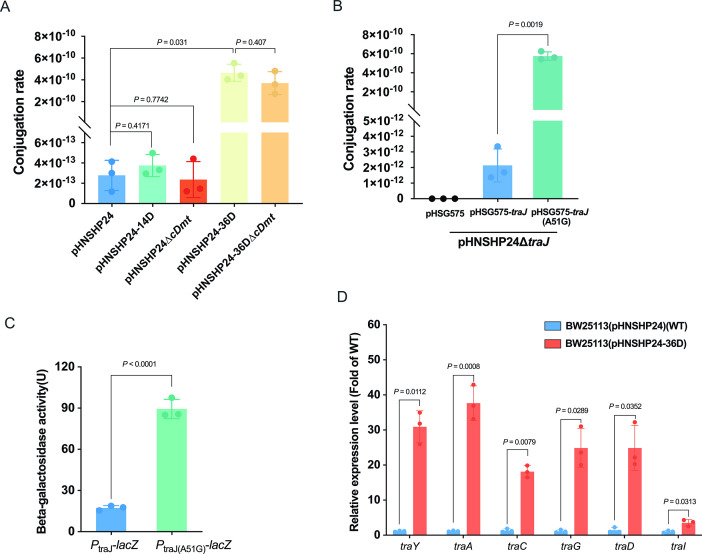
The effect of the A51G mutation located in 5′UTR of *traJ* on the conjugation of pHNSHP24 plasmid. (**A**) Comparison of the conjugation rate of pHNSHP24, pHNSHP24-14D, and pHNSHP24-36D. (**B**) The mutation A51G in 5′UTR of *traJ* promotes the conjugation ability of pHNSHP24. (**C**) Effect of the mutation (A51G) in 5′UTR of *traJ* on the expression of *traJ*. (**D**) Comparison of the transcriptional level of *tra* genes between BW25113(pHNSHP24) and BW25113(pHNSHP24-36D). All plasmid conjugation assays were performed with three biological replicates. One-way analysis of variance was used to determine the statistical differences among groups. The β-Galactosidase assay and qPCR experiment were carried out with three biological replicates, and Student’s *t* test was used for comparison of differences between the two groups.

Given that the mutation A51G in the 5′UTR of *traJ* was responsible for the improvement of plasmid persistence, we investigated the effect of this mutation on conjugation. Conjugation assay was carried out using *E. coli* BW25113 containing pHNSHP24 or the evolved plasmids pHNSHP24-14D and pHNSHP24-36D as donors, and *E. coli* BW25113::kan was used as recipient. As shown in Fig. 3A and Table S1, the transfer rate of pHNSHP24-36D increased ~1,600-fold compared with pHNSHP24 (*P* = 0.031). However, there were no significant differences in the conjugation transfer rate between pHNSHP24 and pHNSHP24-14D (*P* = 0.4171). In addition, we confirmed that the *cDmt* gene has little effect on the conjugation of pHNSHP24 and pHNSHP24-36D plasmids (Fig. 3A and Table S1). This result suggests that the mutation A51G in the 5′UTR of *traJ* led to increased transfer rate. To confirm this finding, we constructed pHNSHP24 ∆ *traJ* and complemented *traJ* (wild type) or mutated *traJ* (A51G) under the control of its native promoter to test their ability to conjugate. As expected, the complementation of mutated *traJ* significantly outperformed the transfer rate of *traJ* (wild type)-complemented pHNSHP24 ∆ *traJ* (Fig. 3B and Table S1). These results indicate that the point mutation A51G in the 5′UTR of *traJ* could indeed increase the plasmid conjugation efficiency.

TraJ is an activator of the *tra* operon (16, 32 - 35). To examine the effect of the mutation A51G on the expression level of *traJ*, we fused the upstream region (−217 to +242) of *traJ* or mutated *traJ* with a promoterless *lacZ*, yielding *P*_traJ_-*lacZ* and *P*_traJ(A51G)_-*lacZ*, respectively. As shown in Fig. 3C, this mutation led to a considerable increase in the β-galactosidase activity, suggesting that this mutation could upregulate the expression of TraJ. Thus, we determined the expression of *tra* genes in both BW25113(pHNSHP24) and BW25113(pHNSHP24-36D) by qPCR. Expectedly, the transcription level of *tra* genes, including *traY*, *traA*, *traC*, *traG*, *traD*, and *traI*, in BW25113(pHNSHP24-36D) strongly increased compared with that of BW25113(pHNSHP24) (Fig. 3D). These results confirmed that the mutation A51G in the 5′UTR of *traJ* increased the plasmid transfer ability by enhancing the expression of *traJ* and *tra* genes.

**Fig 4:**
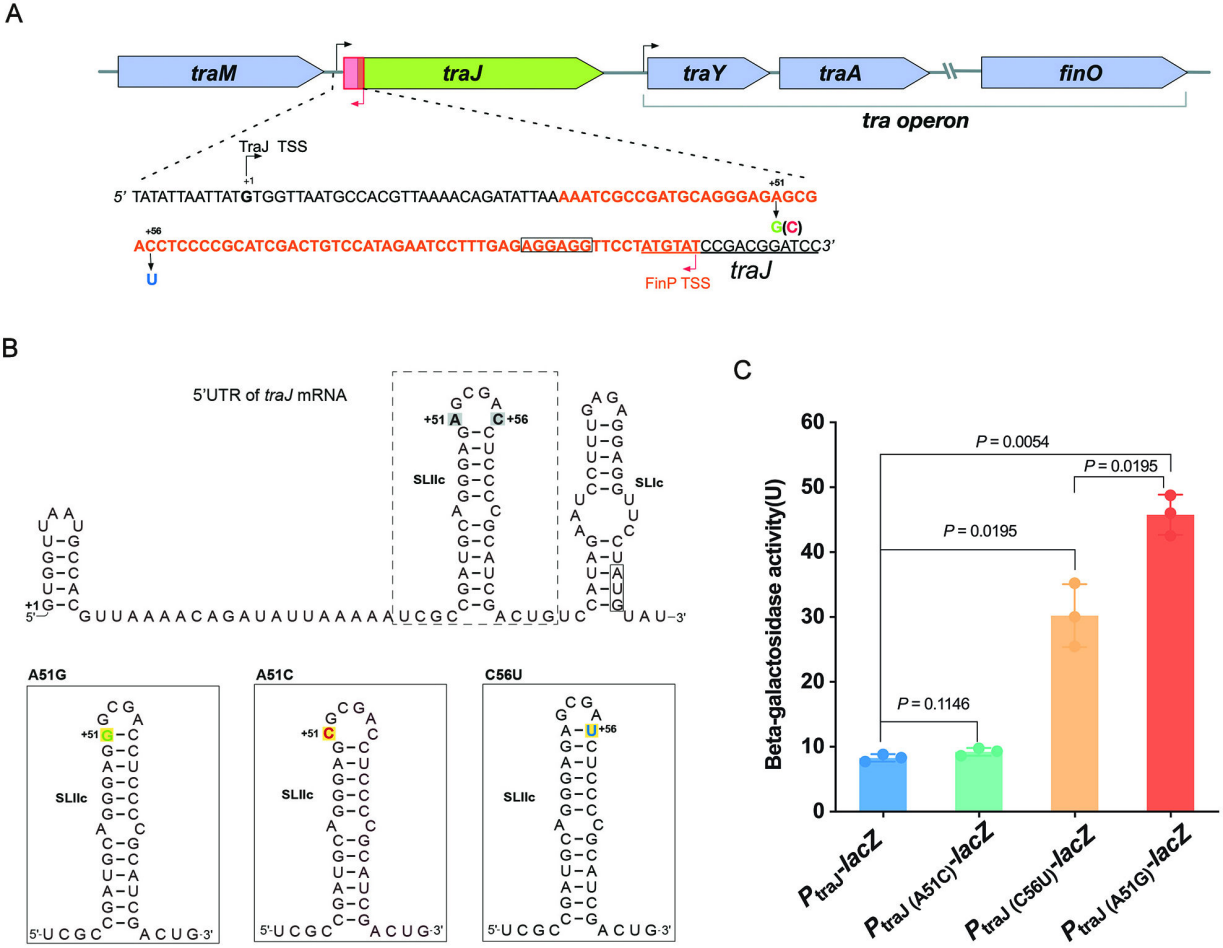
The effect of loop size of SLIIc on the expression of *traJ*. (**A**) Schematic representation of the 5′UTR of *traJ*. The transcription of *traJ* gene starts site (TSS) was denoted as +1. The transcription of FinP RNA starts from the position +107, and its complementary pairing region is displayed by orange fonts. The A51G mutation, A51C mutation, and C56U mutation were marked by green font, red font, and blue font, respectively. The open reading frame of *traJ* was underlined, and the SD sequence of *traJ* was framed by a rectangle. (**B**) The predicted secondary structure of the 5′UTR of *traJ* mRNA. The SLIIc was framed by a dashed rectangle and mutant SLIIc was framed by a solid line rectangle. The loop size of wild-type SLIIc and mutant A51C was 6 nt, while the loop size of the mutants A51G and C56U was 4 nt. (**C**) Effect of the mutations in the loop of SLIIc on the expression of *traJ*. Activity of *traJ* and its derivates (A51C, C56U, and A51G) was monitored from *lacZ* fusion. The β-Galactosidase assay was performed with three biological replicates. One-way analysis of variance was used to determine the statistical differences among different groups.

In IncF plasmids, the expression of *traJ* is negatively regulated by the antisense RNA FinP, which interacts with the leader region of the *traJ* transcript and prevents the translation of *traJ* (Fig. 4A) (36). FinP contains two stem-loop structures, SLI and SLII, which were complementary to the stem-loops of the *traJ* UTR, SLIc, and SLIIc, respectively. Since the point mutation in the 5′UTR of *traJ* (position +51) is located at SLIIc (Fig. 4B), we speculated that this mutation could impact the inhibitory effect of FinP. We predicted the structure of SLIIc of pHNSHP24-36D and found that its loop is smaller than that of pHNSHP24 (Fig. 4B). We hypothesized that the reduced loop of SLIIc impairs the complementary pairing between the 5′UTR of *traJ* mRNA and FinP.

To test the effect of the loop structure of SLIIc on the expression of *traJ*, we changed the loop size of SLIIc by introducing mutations in the 5′UTR of *traJ* (+51 or +56) (Fig. 4B) and fused the upstream region (−217 to +242) with promoterless *lacZ*, yielding *P*_traJ(A51C)_-*lacZ* and *P*_traJ(C56U)_-*lacZ*, respectively. As shown in Fig. 4B, the mutation C56U is predicted to create the same loop size as A51G of pHNSHP24-36D. The β-galactosidase activity of both *P*_traJ(C56U)_-*lacZ* and *P*_traJ(A51G)_-*lacZ* increased significantly compared with *P*_traJ_-*lacZ*(wild-type). Likewise, the mutation A51C is predicted to create the same loop size as in pHNSHP24 and *P*_traJ(A51C)_-*lacZ* shows similar activity to *P*_traJ_-*lacZ* (Fig. 4C). These results suggest that the loop size of SLIIc indeed affects the expression of *traJ*, and the reduction of the loop size caused by the point mutation at 5′UTR of *traJ* (+51) probably impaired the inhibitory effect of FinP on *traJ*. Notably, the *P*_traJ(C56U)_-*lacZ* fusion shows lower activity than *P*_traJ(A51G)_-*lacZ* implying that the stability of SLIIc also affects the inhibitory effect of FinP, since the SLIIc harboring A51G, which contains GC base pair, is probably more stable than the SLIIc harboring C56U, which contains AU base pair.

### The increased plasmid transfer rate accounts for the improved plasmid persistence

**Fig 5:**
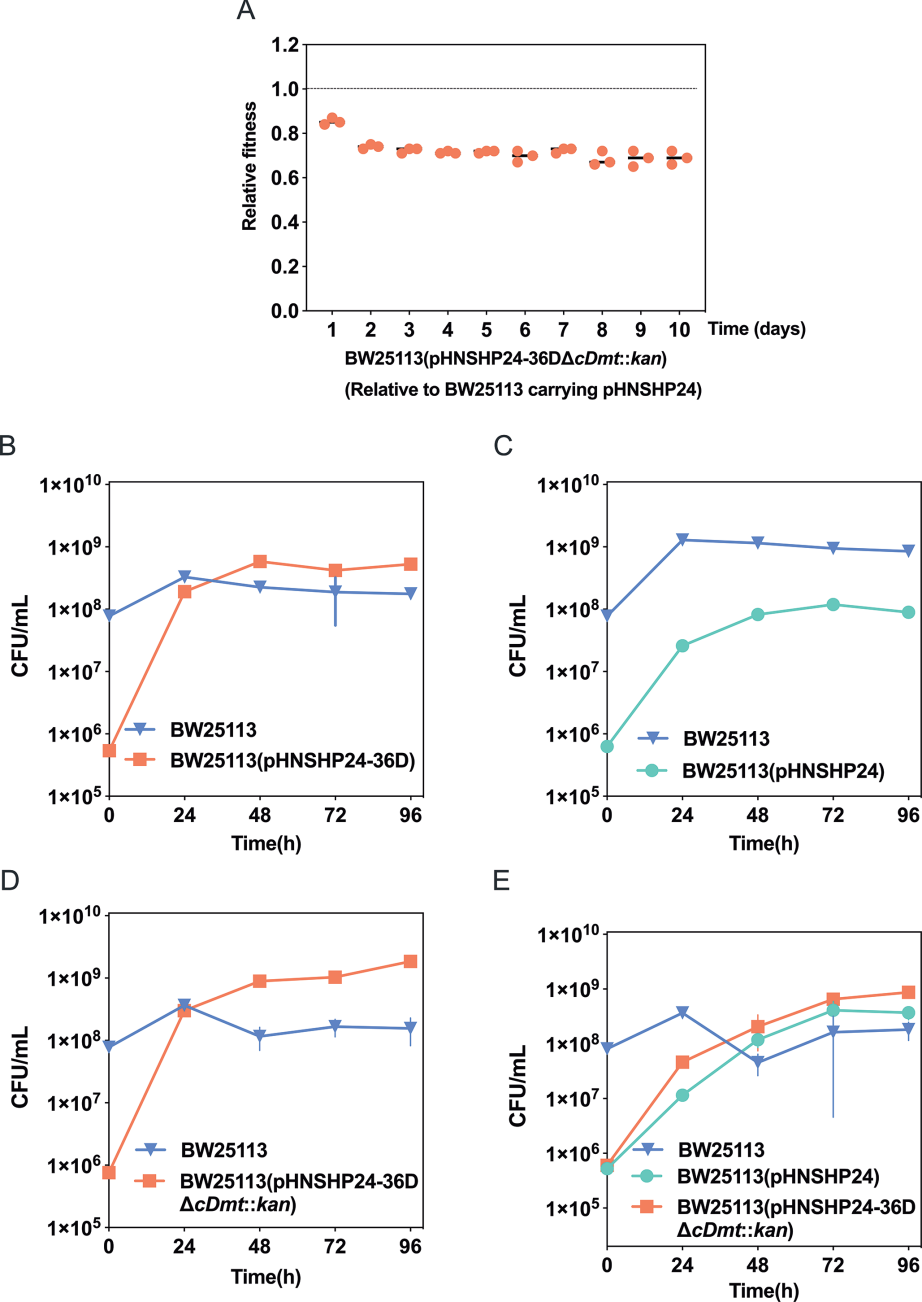
The relative fitness of BW25113(pHNSHP24-36D) (**A**) and bacterial population dynamics in co-cultures with plasmid-free and plasmid-containing *Escherichia coli* BW25113 (**B–E**). Plasmid-containing strains, including BW25113(pHNSHP24), BW25113(pHNSHP24-36D), and BW25113(pHNSHP24-36D ∆ *cDmt::kan*), were mixed with a 1,000-fold excess of *E. coli* BW25113 at the beginning of the invasion assay. *E. coli* BW25113 is indicated by blue triangle, BW25113(pHNSHP24) is indicated by cyan dot, BW25113(pHNSHP24-36D) and BW25113(pHNSHP24-36D ∆ *cDmt::kan*) are indicated by orange square. (**B**) Co-cultures with *E. coli* BW25113 and BW25113(pHNSHP24-36D). (**C**) Co-cultures with *E. coli* BW25113 and BW25113(pHNSHP24). (**D**) Co-cultures with *E. coli* BW25113 and BW25113(pHNSHP24-36D ∆ *cDmt::kan*). (**E**) Co-cultures with *E. coli* BW25113, BW25113(pHNSHP24), and BW25113(pHNSHP24-36D ∆ *cDmt::kan*).

Previous studies have shown that increased plasmid transfer frequency often incurs fitness costs on host bacteria (37, 38). We further compared the costs of pHNSHP24 and pHNSHP24-36D by measuring the growth rates of the plasmid-harboring strains relative to the plasmid-free strain *E. coli* BW25113. As shown in Fig. S1, the growth rates of BW25113 harboring pHNSHP24 and pHNSHP24-36D decreased compared to *E. coli* BW25113, while the growth rate of BW25113(pHNSHP24-36D) was lower than BW25113(pHNSHP24). Then, we also evaluated the fitness of BW25113(pHNSHP24-36D) relative to BW25113(pHNSHP24) in a competition assay. To facilitate the screening of BW25113 harboring pHNSHP24-36D in competition cultures, we introduced a kanamycin resistance gene at the *cDmt* locus since the deletion of *cDmt* in pHNSHP24-36D did not affect fitness (Fig. S2). We used BW25113(pHNSHP24-36DΔ*cDmt*::kan) instead of BW25113(pHNSHP24-36D) for competition assays, since both strains show comparable fitness (Fig. S2). As expected, BW25113(pHNSHP24-36DΔ*cDmt*::kan) showed a significant reduction in fitness relative to BW25113(pHNSHP24) (Fig. 5A), indicating that the evolved plasmid pHNSHP24-36D indeed imposes a higher cost on host bacteria than pHNSHP24. These findings suggest that the evolved 5′UTR of *traJ* increased the plasmid conjugation efficiency but also incurred a higher fitness cost on host bacteria.

Plasmid persistence usually correlates with segregation loss rate (λ), fitness cost (σ) as well as conjugation rate (γ) (21, 31, 39). To investigate how these parameters affect the persistence of plasmids before and after laboratory evolution, we used the plasmid population dynamic model to estimate model-based parameters and evaluate the role of these parameters in plasmid persistence. Initially, to determine the best-fitting model, the plasmid persistence profiles were fitted to both segregation and selection (SS) and the HT models as previously described (21). As shown in Table S2, the HT model provided a better fit, suggesting that conjugation was considered important for the persistence of plasmids in this study. Then, these parameters were estimated by the HT model and listed in Table S3. The maximum likelihood estimates for the segregation rate (λ), ranging from 1.72 × 10^−6^ to 1.99 × 10^−5^, are negligibly low. The model-based conjugation frequency (γ) of BP_E_ (evolved plasmid pHNSHP24-36D) is more than eight times of BP_A_ (ancestral plasmid pHNSHP24); however, its cost is twofold of BP_A_. To better evaluate the role of the model-based conjugation frequency (γ) and cost (σ) on plasmid persistence, we introduced a concept of the persistence threshold γ/θ described by Ponciano et al. (39). θ represents the fraction of the plasmid-carrying cells at which the conjugation frequency is half its maximum. When inequality γ/θ≥1−(1−λ)/2^σ holds, this formula indicates that a high transfer frequency can balance or even offset the frequent loss of plasmids resulting from segregation and fitness cost for the persistence of plasmids in the populations. The γ/θ of BP_E_ satisfied the inequality mentioned above while BP_A_ did not, suggesting that the enhancement of transfer frequency of pHNSHP24-36D compensates for plasmid loss.

We also performed a plasmid invasion experiment to determine the invasion abilities of ancestral and evolved plasmids. The invasion abilities of pHNSHP24-36D and pHNSHP24 were compared individually or in competitive co-cultures. For each plasmid individually, the evolved plasmid pHNSHP24-36D invaded most cells after 24 h (Fig. 5B), indicating that the HT of pHNSHP24-36D was sufficient to offset the segregation loss and fitness cost. In contrast, the ancestral plasmid pHNSHP24 failed to invade the plasmid-free population (Fig. 5C). Given that the deletion of *cDmt* in pHNSHP24-36D did not affect the plasmid transmission ability and fitness cost (Fig. 5D; Fig. S2), we used BW25113(pHNSHP24-36DΔ*cDmt::kan*) instead of BW25113(pHNSHP24-36D) for plasmid invasion in competitive co-cultures. As shown in Fig. 5E, pHNSHP24-36DΔ*cDmt::kan* invaded the cell population faster than pHNSHP24, as expected from the results of the persistence assay.

**Fig 6:**
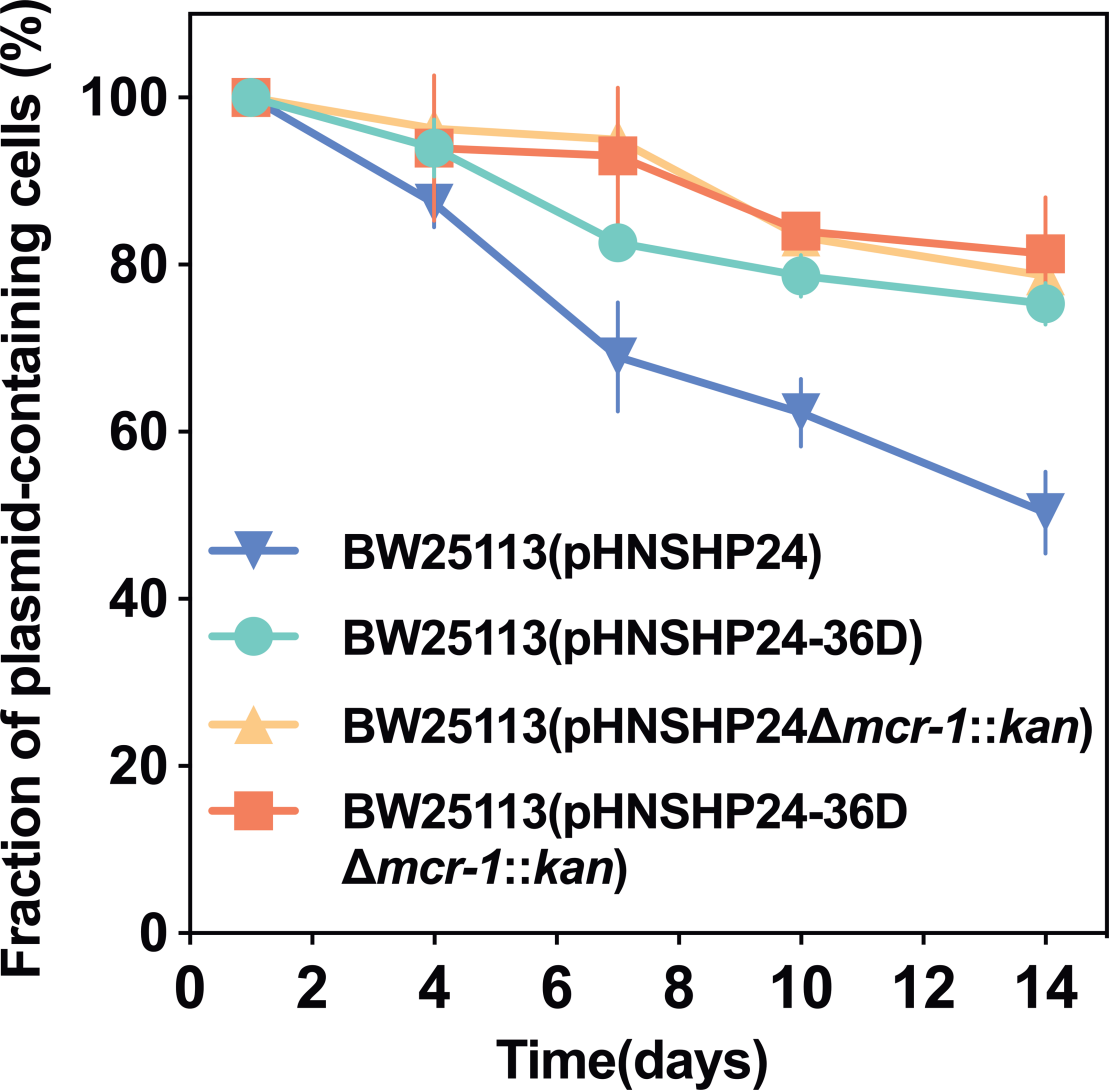
Effect of *mcr-1* on the persistence of pHNSHP24 and pHNSHP24-36D plasmids. Each point represents the mean fraction of plasmid-containing cells (*n* = 3) and bars represent SD of the three biological replicates.

In addition, we investigated the effect of *mcr-1* on plasmid persistence since *mcr-1* usually exerts a fitness cost to host bacteria. We constructed in-frame deletion of *mcr-1* on pHNSHP24 and pHNSHP24-36D and monitored the persistence of pHNSHP24 ∆ *mcr-1::kan* and pHNSHP24-36D ∆ *mcr-1::kan*. Interestingly, the deletion of *mcr-1* improved the persistence of pHNSHP24 considerably, even better than the A51G mutation of pHNSHP24-36D, while the deletion of *mcr-1* slightly improved the persistence of pHNSHP24-36D (Fig. 6). Notably, pHNSHP24 ∆ *mcr-1::kan* and pHNSHP24-36D ∆ *mcr-1::kan* showed very similar maintenance abilities. These findings suggest that the poor persistence of pHNSHP24 is partly due to the cost incurred by *mcr-1*. The evolved 5′UTR of *traJ* improved the persistence of pHNSHP24 in the presence of *mcr-1*; however, without *mcr-1*, it has little effect on plasmid persistence. Altogether, these results confirmed that high transmissibility could offset plasmid loss due to fitness cost and segregation loss under laboratory culture conditions, and pointed to its crucial role in the persistence of *mcr-1*-bearing plasmids.

## DISCUSSION

The plasmid-mediated dissemination of resistance genes has become a great concern to human health (3, 40 - 42). Most antibiotic resistance plasmids not only stably persist in host bacteria for long periods but also confer little fitness cost to host bacteria, implying that these plasmids are well-adapted to the host bacteria (6). However, the evolutionary adaptation of plasmid-bacteria associations is not well understood. Here, we investigated an evolutionary adaptation in laboratory conditions of the persistence of the *mcr-1*-bearing IncFII-pO111 plasmid pHNSHP24 in *E. coli*. We observed that the evolved plasmid had improved persistence in *E. coli*.

In this study, conjugation rate but not fitness cost explained the improved persistence of the evolved plasmid. Although pHNSHP24 contains two putative TA system, *hok*/*sok* and *doc*/*phd*, it still displayed poor persistence in the bacterial populations, suggesting that two putative TA modules may be not involved in post-segregational killing mechanism for maintaining this plasmid. The persistence of the evolved plasmid was improved significantly by the point mutation A51G in the 5′UTR of *traJ*, which boosted the plasmid conjugation rate by ~1,600-fold compared to the ancestral plasmid. However, we observed that the conjugation rate increased at the expense of host fitness. This finding is consistent with the concept that plasmid usually displays a tradeoff between conjugation rate and fitness (37, 38). Since fitness costs produced by plasmids often play a key role in the persistence of plasmid-carrying strains, we evaluated the impact of conjugation rates, fitness cost, and loss rate in the maintenance of the evolved plasmid by the plasmid population dynamic HT model. We found that, under laboratory conditions, an increase in conjugation rate improved the persistence of pHNSHP24. The plasmid invasion experiment also showed that the increased transfer rate of pHNSHP24-36D could compensate for the plasmid fitness cost. The evolved plasmid was more stable in host bacteria, presumably because more conjugation meant plasmid-free bacteria were more likely to be reinfected.

Previous observations of plasmid evolution showed that compensatory evolution could increase plasmid persistence significantly, often by reducing the fitness cost of a costly plasmid-bacteria association (18, 19, 21). Furthermore, compensatory mutations can also target the transfer region and inhibit or abolish conjugation (20, 43). Unexpectedly, we observed that the *mcr-1* plasmid pHSNHP24 improved its persistence by the evolution of conjugation transfer, even though the evolved plasmid incurred a higher fitness cost than the ancestral plasmid. Deletion of *mcr-1* could enhance the plasmid persistence to some extent, suggesting that the plasmid cost on the host partly resulted from *mcr-1*. These observations are consistent with our previous study, which suggests that efficient conjugative transfer is crucial for *mcr-1*-bearing IncX4 plasmid to alleviate fitness cost and promote its persistence in the bacterial populations (25). Although the evolved infectious transmission favors the maintenance of the unstable *mcr-1*-bearing plasmid pHNSHP24, this evolution has little effect on the persistence of *mcr-1*-deficient plasmid pHNSHP24 ∆ *mcr-1*. These results reflect that, in addition to low copy number (44), high conjugation rate may be another important contributor to the maintenance of *mcr-1* plasmids. A limitation of our study is that the plasmid evolution and persistence experiment were conducted in the laboratory environment which is very different from the natural environment. The plasmid transfer and persistence in the natural microbial communities are very complex. The species diversity and interspecies interactions could impact the plasmid dynamics in these communities (45). For example, bacteriophages and protist predation could limit conjugative plasmid persistence by affecting the microbial community structure (45). Less permissive species may hinder the spread of conjugative plasmids in communities, and diversity in fitness effects of plasmids within different species may facilitate the persistence of costly plasmids in bacterial communities (45). In addition, since the host bacteria used for the evolution of pHNSHP24 plasmid was not the natural host of this plasmid, the difference in genetic background of host bacteria may also influence the evolution of this plasmid.

The FinOP system that regulates the expression of the *tra* operon in IncF plasmids has been investigated in detail (46). The antisense RNA FinP represses TraJ translation by pairing with its *traJ* mRNA target, facilitated by FinO (47, 48). Pairing between FinP and *traJ* mRNA is thought to initiate between complementary loops in the two RNAs (49). Here, we show that point mutations located in *traJ* 5′UTR that reduce the predicted loop size of SLIIc from 6 to 4 nt negatively affect the inhibitory effect of FinP on *traJ*. This finding is reminiscent of the binding reaction between CopA and CopT RNAs of plasmid R1. Likewise, CopA/CopT-binding process initiates with the formation of a transient “kissing complex” between two RNAs (50). The size alterations of CopA loop II have been shown to strongly affect the binding rates of CopA and CopT (51). Notably, the most efficient binding rates for the two RNAs were obtained with loop II of 6–7 unpaired nucleotides, while small (4 nt) loop II led to significantly decreased binding rates and, hence, the inhibitory effect of CopA on CopT (51). We propose that mutations reducing the loop size of SLIIc impair the binding rates between FinP and 5′UTR of *traJ* mRNA and therefore decrease the inhibitory effect of FinP on its target. It should be noted that the loop size of the C56U mutant was 4 nt, which is consistent with that of the A51G mutant. However, the C56U mutant shows lower expression level of *traJ* than that of the A51G mutant. The C56U mutant contains an AU base pair, while the A51G mutant contains a GC base pair that is the only difference between them, indicating that the stability of SLIIc also affects the inhibitory effect of FinP on *traJ*. In fact, the 5′UTR of *traJ* from various F-like plasmids contain highly conserved stems of SLIIc structure but various loop regions (52) , suggesting that various loop regions of SLIIc may represent one evolutionary strategy for F-like plasmids transfer. In addition, the amino acid sequence of TraJ from F-like plasmids varies greatly, and the promoter sequences of *tra* operon which is activated by TraJ are also differential (16). It is worth noting that some F-like plasmids could co-exist in the same host bacterium without interference in regulation of plasmid transfer (16), indicating that F-like plasmids have evolved specialized transfer regulatory systems.

In conclusion, we showed that long-term positive selection on *mcr-1*-bearing plasmid pHNSHP24 can lead to the evolution of conjugation and an increased conjugation transfer rate could enhance its maintenance in pure culture conditions. The loop size and stability of SLIIc in 5′UTR of *traJ* can influence the transfer rate of pHNSHP24. The plasmid conjugative transfer is important for maintaining the *mcr-1*-bearing plasmid. Our findings emphasize that inhibition of the conjugation process could be another approach apart from the plasmid-curing strategy to combat the spread and persistence of antibiotic-resistance plasmids.

## MATERIALS AND METHODS

### Bacterial strains and growth conditions

The bacterial strains used in this study are listed in Table S4. All bacterial strains were cultured in LB broth at 37°C with shaking (180 rpm) or on LB agar plates at 37°C. Strains containing pHSG575 plasmid and its derivatives were cultured in LB broth supplemented with chloramphenicol (30 μg/mL), and strains containing pHGR01 plasmid and its derivatives were cultured in LB broth supplemented with kanamycin (30 μg/mL). The strain *E. coli* BW25113 and transconjugants carrying pHNSHP24 or pHNSHP24-36D were grown in LB broth overnight at 37°C, then diluted 1:100 into 100 mL fresh medium and shaking (180 rpm) at 37°C for 12 h. Bacterial growth was measured OD_600_ every hour by Multiskan spectrum microplate spectrophotometer (Thermo Labsystems, Franklin, MA, USA).

### Experimental evolution and plasmid persistence experiment

The *E. coli* C600 harboring pHNSHP24 plasmid (GenBank accession number: CP065023) was cultured serially for 36 days in the presence of colistin (2 μg/mL). The *E. coli* C600(pHNSHP24) strain was grown in LB broth containing colistin overnight at 37℃, then 3μL culture was transferred into 3 mL LB broth containing colistin and shaken at 37℃ each day. The plasmid persistence was measured following 7, 14, and 36 days of serial transfers. Briefly, the evolved cultures or strains were cultured serially in the absence of colistin for 14 days. The cultures which were passaged for 4, 7, 10, and 14 days were spread on non-selective LB agar plates with proper dilution, then 50 colonies were randomly selected to confirm the presence of plasmid by PCR using primers FrepB-F/FrepB-R, mcr-1-F/mcr-1-R, and pO111-F/pO111-R (Table S5).

### Comparison of plasmid persistence

The plasmid persistence profile describes the plasmid persistence over time (from days 0 to 10). The plasmid persistence profile was analyzed and compared using plasmid population dynamics models as described previously (21, 31). The two models named SS model or HT model, describe the plasmid dynamics based on maximum likelihood estimates (MLEs) of the segregation rate (λ), conjugation rate (γ), and cost (σ). The SS model involves only two parameters, λ and σ, but the HT model needs all parameters. Initially, plasmid persistence profiles were fit to both models to find the best-fitting model. The best-fitting model is reflected by the lowest negative log-likelihood score. Then, different profiles were compared with each other using the best-fitting model. The BIC reflects the difference between two persistence profiles (the smaller the ΔBIC values, the greater difference between two plasmid persistence dynamics) (31). Moreover, we use the HT model to estimate plasmid cost, segregational loss frequencies, and conjugation frequency based on plasmid persistence profiles. A formula described by Ponciano et al. was used to reflect the effect of conjugation, cost, and segregation on plasmid persistence (39).


$$\frac{\gamma }{\theta }\geq 1-\frac{1-\lambda }{2^{\sigma }}$$

where γ is an asymptotic maximum conjugation frequency within a period of time, and θ represents the fraction of the plasmid-carrying cells when the frequency of conjugations is half the maximum. γ/θ represents the transfer frequency threshold needed to ensure the persistence of plasmids. *λ* is segregational loss frequency and σ is plasmid cost. This formula states that plasmid loss due to segregation and cost must be balanced by a high transfer frequency for the plasmids to persist in the populations. It should be noted that γ described here is a model-based conjugation transfer frequency, and it is not the same as the experimental estimated transfer rate γ estimated by the conjugation experiment based on the Simonsen method. All these analyses were conducted using the R package “StabilityToolkit” (https://github.com/jmponciano/StabilityToolkit/blob/master/RunningStabToolsPack.zip) created by Ponciano et al. (39).

### Plasmid sequencing and analysis

The whole genomes of the clones that evolved for 36 days (E1-1, E1-2, and E1-3) and clones that evolved for 7 and 14 days in E1 lineage were extracted and subjected to perform whole-genome sequencing (Illumina, San Diego, CA, USA) and Nanopore sequencing (Oxford Nanopore Technologies, UK). Nanopore reads and Illumina reads were combined to produce a *de novo* hybrid assembly using Unicycler version 0.4.3. The plasmid sequence comparison was performed using BLAST.

### Construction of plasmids and strains

Plasmids used in this study are listed in Table S4, and all primers are listed in Table S5. The fragments of *traJ* with their native promoter were amplified from pHNSHP24 and pHNSHP24-36D using primers pro-traJ-F/pro-traJ-R, respectively, and cloned into pHSG575 to generate pHSG575-traJ and pHSG575-traJ(A51G). The upstream region of *traJ* with its native promoter (−217 to +242) was amplified from pHNSHP24 or pHNSHP24-36D using primers *lacZ*-traJ-F*/lacZ*-traJ-R and cloned into the HindIII site of pHGR01 plasmid which contains a promoterless *lacZ* gene to produce *P*_traJ_-*lacZ* and *P*_traJ(A51G)_-*lacZ*. The *traJ* (A51C) mutation in *P*_traJ_-*lacZ* was constructed by *lacZ*-traJ-F*/*mu-24traJ(A51C)-R primers and mu-24traJ(A51C)-F/*lacZ*-traJ-R primers. The *traJ*(C56U) mutation in *P*_traJ_-*lacZ* was constructed by *lacZ*-traJ-F*/*mu-24traJ(C56U)-R primers and mu-24traJ(C56U)-F/*lacZ*-traJ-R. Deletion mutants of pHNSHP24 and pHNSHP24-36D were constructed using the λ Red recombination system (53). Deletion of the *traJ* gene was done on pHNSHP24 using primers knock-traJ-F/knock-traJ-R and check-traJ-F/check-traJ-R. Deletion of the *cDmt* gene was done on pHNSHP24-36D and pHNSHP24 using primers knock-cDmt-F/knock-cDmt-R and check-cDmt-F/check-cDmt-R.

The kanamycin-resistant gene was inserted in the truncated *lacZ* gene of *E. coli* BW25113 to construct the kanamycin-resistant *E. coli* BW25113::kan using λ Red recombination system. The kanamycin-resistant gene fragment with homology extension was cloned from the pKD4 plasmid using primers insert-kan-F/insert-kan-R. The mutant was verified by PCR using primers check-kan-F/check-kan-R.

### Bacterial conjugation assays

The conjugation experiments were set up based on the Simonsen method (54) to estimate the plasmid conjugation rate ( γ ). All plasmid conjugation assays were performed with three biological replicates. The colistin-resistant strains BW25113(pHNSHP24) and its derivatives were used as donors (D), and the kanamycin-resistant *E. coli* BW25113::kan was used as recipient (R). Thus, donors (D), recipients (R), and transconjugants (T) could be estimated by selective LB plates containing colistin (2 μg/mL) and/or kanamycin (30 μg/mL). The donor and recipient were individually cultured in 5 mL LB broth overnight at 37℃. The overnight cultures were adjusted to the same density (OD_600_=1), and 3 µL of each culture was transferred into tubes containing 3 mL LB broth. At the start point of mating, 100 µL mixture was sampled to quantify the total initial cell densities (*N*_0_). The mating cultures were incubated at 37℃ with shaking for 12 h. During the exponential growth phase, the optical density was measured at *t*_b_ and *t*_a_ to calculate the growth rate of the mating cultures, using the following formula (54):


$$\psi =\frac{\ln\left({OD}_{b}/{OD}_{a}\right)}{t_{b}-t_{a}}$$

where ψ is the growth rate of the mating cultures, ${OD}_{b}$ and ${OD}_{a}$ represent the optical density at *t*_b_ and *t*_a_, respectively. This model assumes that donors (D), recipients (R), and transconjugants (T) have the same growth rate. However, if they do not have the same growth dynamics, it could lead to biased estimates (54, 55).

At the endpoint time (12 h) of mating, the mating cultures with proper dilution were spread on selective LB plates to quantify the total cell densities (N), donors (D), recipients (R), and transconjugants (T). The mating cultures with serial dilutions (1:10^5^, 1:2 × 10^5^, and 1:4 × 10^5^) were plated to colistin-containing and kanamycin-containing plates. The mating cultures with serial dilutions (1:10, 1:10^2^, 1:10^3^, 1:10^4^, 1:10^5^, 1:2 × 10^5^, and 1:4 × 10^5^) were plated to plates containing both colistin and kanamycin. The number of donors (D) was calculated by the following formula: donors (D)=colistin-containing plate counts minus two antibiotics-containing plate counts. The number of recipients (R) was calculated by the following formula: recipients (R)=kanamycin-containing plate counts minus two antibiotics-containing plate counts. The number of transconjugants (T) was counted from two antibiotics-containing plates. The total cell densities (N)=donors (D)+recipients (R)+transconjugants (T). The transfer rate ( γ ) was calculated using formula (54):


$$\gamma =\psi ln\left(1+\frac{T}{R}\frac{N}{D}\right)\frac{1}{N-N_{0}}$$

### Competition experiments *in vitro* and plasmid invasion assays

Competition experiments were used to measure the relative fitness of strains harboring pHNSHP24 or its derivatives. The overnight cultures of the two strains were diluted 1:1,000 in LB broth and mixed at a 1:1 ratio. The mixture was incubated at 37°C with shaking (180 rpm) for 24 h and then the mixed population was again 1:1,000 diluted into fresh LB broth. This procedure was repeated until the competition experiment lasted for 4 days. The samples of each competitive mixture with proper dilution were spread on LB agar every 24 h. The properly diluted cultures were plated to antibiotic-free, kanamycin-containing, or colistin-containing LB agar plates. The formula RF= log_10_ S1_dt_–log_10_ S1_d0_)/(log_10_ S2_dt_–log_10_ S2_d0_) was used to calculate relative fitness (RF), where RF is the relative fitness of S1 strain (compared to S2 strain), S1_dt_ and S1_d0_ represent the densities of cells (CFU/mL) of S1 and the end and beginning of the competition, and S2_dt_ and S2_d0_ represent the densities of cells of S2 and the end and beginning of the competition. All competition experiments were carried out with three biological replicates.

Plasmid invasion assays were performed according to the method described in the previous study (56). All plasmid invasion assays were performed with three biological replicates. Overnight cultures of BW25113 were diluted 1:10 into 2 mL LB broth and mixed with 1:1,000 dilutions of BW25113(pHNSHP24), BW25113(pHNSHP24-36D), or BW25113(pHNSHP24-36DΔ*cDmt::kan*) overnight cultures, respectively. The 2 mL mixed cultures grew in a 50-mL tube at 37℃ with shaking (45 rpm), and diluted 1:100 into fresh media every 24 h for 96 h. Viable counts were performed at 24, 48, 72, and 96 h. For each time point, dilutions of the cultures were plated to non-selective, colistin-containing and kanamycin-containing media. Cells containing pHNSHP24 or pHNSHP24-36D grew on non-selective and colistin-containing plates but were killed by kanamycin-containing media. Cells containing pHNSHP24-36DΔc*Dmt::kan* grew on all plates. Plasmid-free cells were killed by both antibiotics. Therefore, the number of cells containing pHNSHP24, pHNSHP24-36D, and pHNSHP24-36DΔc*Dmt::kan* were counted from colistin-containing plates, colistin-containing plates, and kanamycin-containing plates, respectively. The number of plasmid-free cells BW25113 was calculated by the following formula: BW25113 (plasmid-free) cells=antibiotic-free plate counts minus colistin-containing/kanamycin-containing plate counts. The experimental procedure for a mixture containing BW25113, BW25113(pHNSHP24), and BW25113(pHNSHP24-36DΔc*Dmt::kan*) is consistent with the steps described above. The cell numbers of three strains were calculated by the following formula: BW25113 (plasmid-free) cells=antibiotic-free plate counts minus colistin-containing plate counts, BW25113(pHNSHP24) cells=colistin-containing plate counts minus kanamycin-containing plate counts, BW25113(pHNSHP24)-36DΔc*Dmt::kan* cells=kanamycin-containing plate counts.

### RNA extraction, cDNA synthesis, and qPCR

Equal numbers of bacteria during the log phase of growth were collected and total RNA was extracted using E.Z.N.A. Bacterial RNA Kit (OMEGA BIO-TEK, USA), according to the product manual. Then, the integrity of total RNA was confirmed by agarose gel electrophoresis and quantified by NanoDrop 2000c. The RNA samples with 23S/16S rRNA ratio≈2.0 and the *A*_260_/*A*_280_ between 1.8 and 2.2 were used for cDNA synthesis. About 1 µg total RNA was used for reverse transcription using Goldenstar RT6 cDNA Synthesis Mix (TsingKe Biotech, China), according to the production protocol. qPCR was performed using the TB Green Premix Ex Taq II reagent (TaKaRa, Japan) and Bio-Rad CFX96 Real-Time PCR Detection System (Bio-Rad, USA). The reaction mixture contained 10 µL of TB Green Premix Ex Taq II, 1 µL of cDNA (diluted 1:10), 0.5 µL of each primer (10 µM), and 8 µL of double distilled water. The amplification was performed as the following conditions: 95℃ for 5 min, 40 cycles of 95℃, 30 s; 60℃, 30s; 72℃, 20s, followed by the melting reaction from 65℃ to 95℃. The relative gene expression levels were calibrated by the 2^−ΔΔCt^ method and *gapA* gene was used as reference gene. The tenfold gradient-diluted cDNA mixture from all samples was amplified for construction of standard curve. The standard curve was used for calculating the amplification efficiency (E) and correlation coefficient values (*R*^2^) of the primers (57). The amplification efficiency (E) for each pair of primers ranges from 90% to 110%, and the correlation coefficient values (*R*^2^) range from 0.98 to 1. The relative expression level of genes was obtained by the 2^−ΔΔCt^ method (58). All experiments were carried out in three biological replicates and two technical replicates.

### β-Galactosidase assay

Cells carrying *lacZ* fusion vectors were cultured in LB broth containing kanamycin overnight at 37℃. Overnight cultures diluted 1:10 into fresh media with shaking (180 rpm) at 37℃ for 4 h. Cells were collected and resuspended in lysis buffer (50 mM Tris–HCl pH 7.3, 1 mM DTT, 5% glycerol, and 1 mM EDTA) and lysed by sonication. The lysates were centrifuged at 12,000× *g* for 10 min at 4℃ to obtain supernatants, adding 4 μL of the supernatant to Bradford Protein Assay Kit (TaKaRa, Japan) to determine protein concentration. About 50 μL of the supernatant was taken to mix with 950 μL of the lysis buffer. Whereafter, recording began immediately after the addition of 0.2 mL of *o*-nitrophenyl-β-D-galactopyranoside (4 mg/L), with all reactions performed at room temperature. Reactions were terminated when the color of mixtures turned yellow by the addition of 500 μL of 1 M Na_2_CO_3_. The optical density at 420 nm (OD_420_) was measured by Multiskan spectrum microplate spectrophotometer (Thermo Labsystems, Franklin, MA, USA). The units of activity were calculated as previously described (59).

## Data Availability

The sequence data in this study have been submitted to the Sequence Read Archive (SRA) in National Center for Biotechnology Information under accession number PRJNA936493 . The authors declare that all data supporting the findings of this study are available within the article and the Supplementary data.

## SUPPLEMENTAL MATERIAL

Fig. S1 mbio.00442-23-s0001.docx The relative growth rate of *E. coli* BW25113(pHNSHP24) and BW25113(pHNSHP24-36D). The growth rate of BW25113(pHNSHP24), BW25113(pHNSHP24-36D) and plasmid-free cells BW25113 were measured as the increase in OD_600_ per hour during exponential growth phase.

Fig. S2 mbio.00442-23-s0002.docx Effect of *cDmt* on bacterial fitness.

Table S1 mbio.00442-23-s0003.docx The colony counts of mating cultures at the end-point of mating assay.

Table S2 mbio.00442-23-s0004.docx S2 BIC (the estimated negative log-likelihood score) for both model (SS and HT model) based on the plasmid persistence profile for *E. coli* BW25113.

Table S3 mbio.00442-23-s0005.docx Estimated segregational loss frequencies (λ), cost (σ), conjugation frequency (γ) and θ for *E. coli* BW25113 carrying ancestral, evolved plasmid and variants using horizontal transfer (HT) model.

Table S4 mbio.00442-23-s0006.docx Bacterial strains and plasmids used in this study.

Table S5 mbio.00442-23-s0007.docx Primers used in this study.

ASM does not own the copyrights to Supplemental Material that may be linked to, or accessed through, an article. The authors have granted ASM a non-exclusive, world-wide license to publish the Supplemental Material files. Please contact the corresponding author directly for reuse.
