# Supplementary material for: The evolution of infectious transmission promotes the persistence of mcr-1 plasmids
Source: mBio. 2023 Jun 14;14(4):e00442-23. doi: 10.1128/mbio.00442-23 (PMC10470590; doi:10.1128/mbio.00442-23)
Supplement: Fig. S1 — The relative growth rate of E. coli BW25113(pHNSHP24) and BW25113(pHNSHP24-36D). The growth rate of BW25113(pHNSHP24), BW25113(pHNSHP24-36D) and plasmid-free cells BW25113 were measured as the increase in OD600 per hour during exponential growth phase. [file mbio.00442-23-s0001.docx]

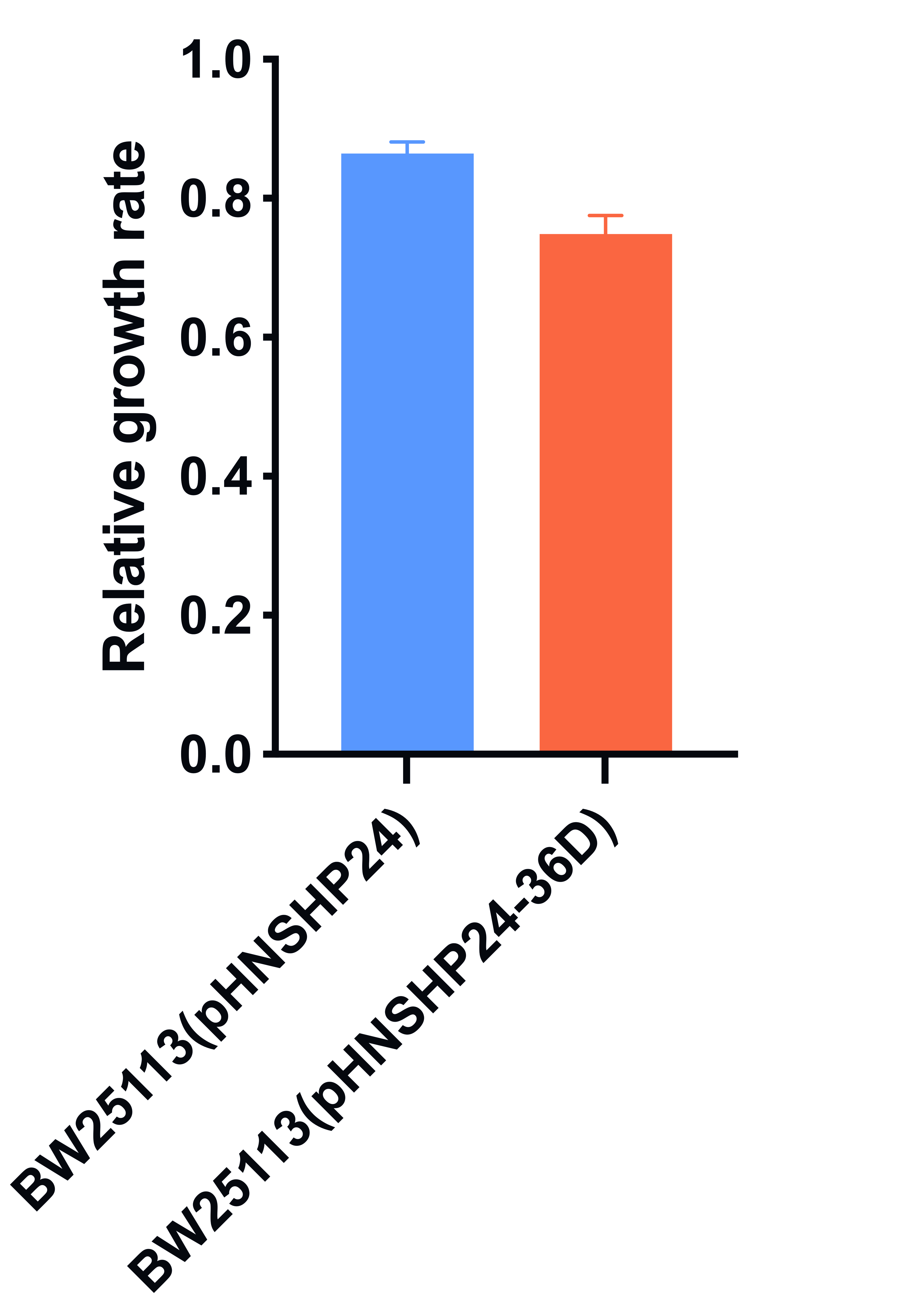


**Fig S1** The relative growth rate of *E. coli* BW25113(pHNSHP24) and BW25113(pHNSHP24-36D). The growth rate of BW25113(pHNSHP24), BW25113(pHNSHP24-36D) and plasmid-free cells BW25113 were measured as the increase in OD_600_ per hour during exponential growth phase. The relative growth rate of each strain is a ratio relative to BW25113. Each point represents the mean of three biological replicates and error bars represent the SD.
