# Supplementary material for: The evolution of infectious transmission promotes the persistence of mcr-1 plasmids
Source: mBio. 2023 Jun 14;14(4):e00442-23. doi: 10.1128/mbio.00442-23 (PMC10470590; doi:10.1128/mbio.00442-23)
Supplement: Fig. S2 — Effect of cDmt on bacterial fitness. [file mbio.00442-23-s0002.docx]

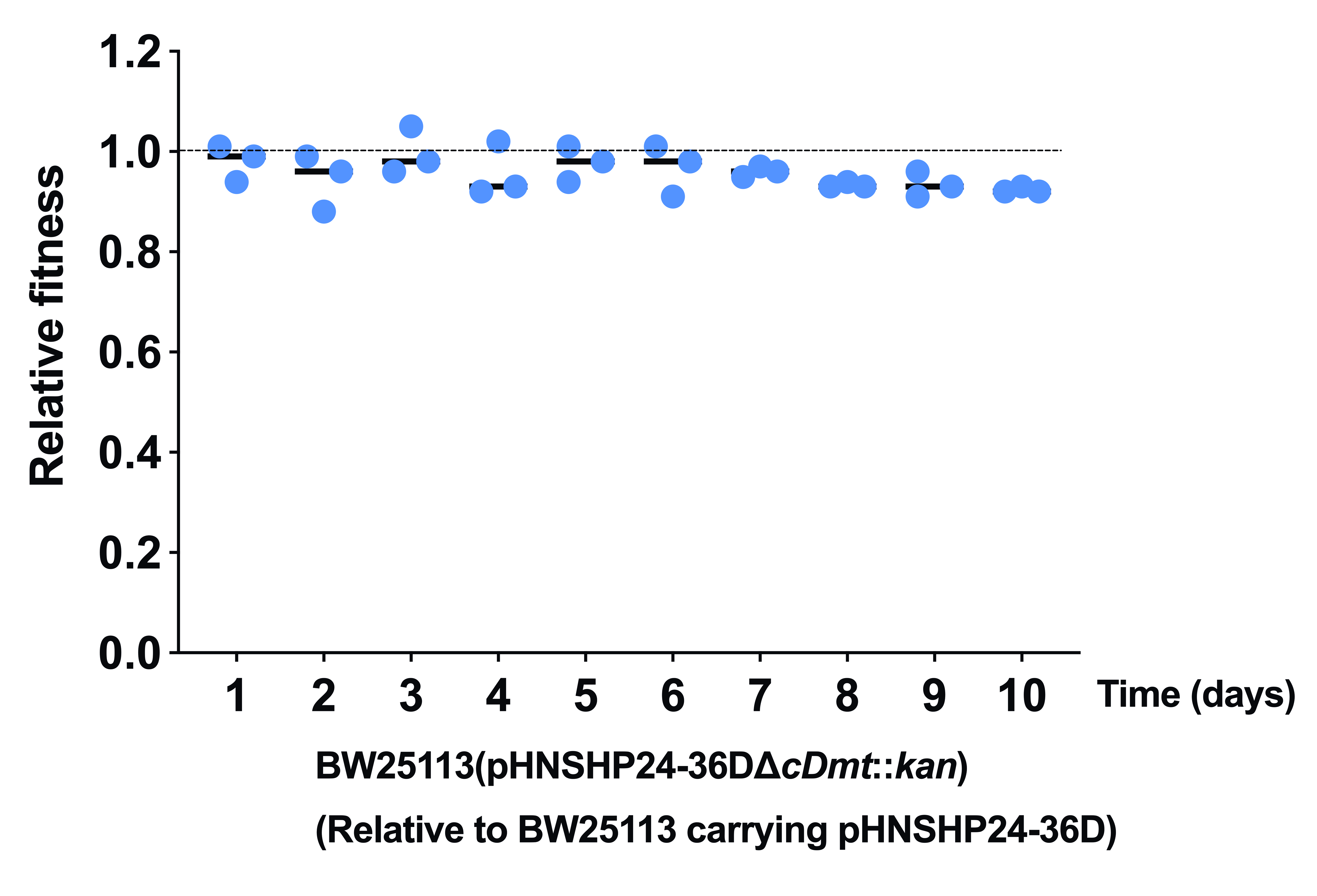


**Fig S2** Effect of *cDmt* on bacterial fitness. BW25113(pHNSHP24-36D∆c*Dmt*::*kan*) was competed with BW25113(pHNSHP24-36D). The competition assay was carried out with three replicates and last for 10 days.
