# Supplementary material for: The evolution of infectious transmission promotes the persistence of mcr-1 plasmids
Source: mBio. 2023 Jun 14;14(4):e00442-23. doi: 10.1128/mbio.00442-23 (PMC10470590; doi:10.1128/mbio.00442-23)
Supplement: Table S1 — The colony counts of mating cultures at the end-point of mating assay. [file mbio.00442-23-s0003.docx]

**Table S1 The colony counts of mating cultures at the end-point of mating assay**

| Colony numbers on each selective plate^a^ | | | | | | | | | | | |
| --- | --- | --- | --- | --- | --- | --- | --- | --- | --- | --- | --- |
|  | **Independent repeat1** | | |  | **Independent repeat2** | | |  | **Independent repeat3** | | |
| Plates containing kanamycin (Kan) and/or colistin (Cl) | **Kan^R^** | **Cl^R^** | **Kan+Cl^R^** |  | **Kan^R^** | **Cl^R^** | **Kan+Cl^R^** |  | **Kan^R^** | **Cl^R^** | **Kan+Cl^R^** |
| pHNSHP24 | 291（4$\times$10^5^） | 156（4$\times$10^5^） | 129（1$\times$10^3^） |  | 231（4$\times$10^5^） | 128（4$\times$10^5^） | 70（1$\times$10^3^） |  | 238（4$\times$10^5^） | 170（4$\times$10^5^） | 37（1$\times$10^3^） |
| pHNSHP24-14D | 249（4$\times$10^5^） | 191（4$\times$10^5^） | 134（1$\times$10^3^） |  | 197（4$\times$10^5^） | 231（4$\times$10^5^） | 105（1$\times$10^3^） |  | 174（4$\times$10^5^） | 186（4$\times$10^5^） | 120（1$\times$10^3^） |
| pHNSHP24$\boldsymbol{\Delta}$*cDmt* | 152（4$\times$10^5^） | 167（4$\times$10^5^） | 27（1$\times$10^3^） |  | 80（4$\times$10^5^） | 84（4$\times$10^5^） | 23（1$\times$10^3^） |  | 180（4$\times$10^5^） | 171（4$\times$10^5^） | 33（1$\times$10^3^） |
| pHNSHP24-36D | 74（2$\times$10^5^） | 220（4$\times$10^5^） | 64（2$\times$10^5^） |  | 58（2$\times$10^5^） | 193（4$\times$10^5^） | 44（2$\times$10^5^） |  | 61（2$\times$10^5^） | 281（4$\times$10^5^） | 52（2$\times$10^5^） |
| pHNSHP24-36D$\boldsymbol{\Delta}$*cDmt* | 54（2$\times$10^5^） | 201（4$\times$10^5^） | 42（2$\times$10^5^） |  | 49（2$\times$10^5^） | 212（4$\times$10^5^） | 41（2$\times$10^5^） |  | 86（2$\times$10^5^） | 180（4$\times$10^5^） | 47（2$\times$10^5^） |
| pHNSHP24$\boldsymbol{\Delta}$traJ/pHSG575 | 260（4$\times$10^5^） | 260（4$\times$10^5^） | 0（0） |  | 204（4$\times$10^5^） | 106（4$\times$10^5^） | 0（0） |  | 235（4$\times$10^5^） | 116（4$\times$10^5^） | 0（0） |
| pHNSHP24$\boldsymbol{\Delta}$traJ/pHSG575  -traJ | 109（4$\times$10^5^） | 338（4$\times$10^5^） | 368（1$\times$10^3^） |  | 61（4$\times$10^5^） | 153（4$\times$10^5^） | 239（1$\times$10^3^） |  | 81（4$\times$10^5^） | 211（4$\times$10^5^） | 197（1$\times$10^3^） |
| pHNSHP24$\boldsymbol{\Delta}$traJ/pHSG575  -traJ(A51G) | 61（2$\times$10^5^） | 247（4$\times$10^5^） | 52（2$\times$10^5^） |  | 60（2$\times$10^5^） | 275（4$\times$10^5^） | 53（2$\times$10^5^） |  | 55（2$\times$10^5^） | 234（4$\times$10^5^） | 48（2$\times$10^5^） |

^a^The colistin-resistant strains BW25113(pHNSHP24) and its derivatives were used as donors(D), and the kanamycin-resistant *E. coli* BW25113::kan was used as recipient (R). The transconjugants(T) were resistant to colistin and kanamycin. All plasmid conjugation assays were performed with three biological replicates.

The mating cultures (end-point) with proper dilution (50 μl) were plated to different selective plates for colony counts. The dilution multiple of each mating culture is indicated in parenthesis.
