# Supplementary material for: The evolution of infectious transmission promotes the persistence of mcr-1 plasmids
Source: mBio. 2023 Jun 14;14(4):e00442-23. doi: 10.1128/mbio.00442-23 (PMC10470590; doi:10.1128/mbio.00442-23)
Supplement: Table S2 — S2 BIC (the estimated negative log-likelihood score) for both model (SS and HT model) based on the plasmid persistence profile for E. coli BW25113. [file mbio.00442-23-s0004.docx]

**Table S2** BIC (the estimated negative log-likelihood score) for both model (SS and HT model) based on the plasmid persistence profile for *E. coli* BW25113 carrying the ancestral and evolved plasmid.

| Strains^b^ | BIC^a^ SS | BIC^a^ HT |
| --- | --- | --- |
| BP_A_ | 167 | 132 |
| BP_E_ | 124 | 105 |

^a^ The best fitting model is reflected by the lowest negative log likelihood score. ^b^ BP_A_ represents BW25113(pHNSHP24); BP_E_ represents BW25113(pHNSHP24-36D).
