# Supplementary material for: The evolution of infectious transmission promotes the persistence of mcr-1 plasmids
Source: mBio. 2023 Jun 14;14(4):e00442-23. doi: 10.1128/mbio.00442-23 (PMC10470590; doi:10.1128/mbio.00442-23)
Supplement: Table S3 — Estimated segregational loss frequencies (λ), cost (σ), conjugation frequency (γ) and θ for E. coli BW25113 carrying ancestral, evolved plasmid and variants using horizontal transfer (HT) model. [file mbio.00442-23-s0005.docx]

**Table S3** Estimated segregational loss frequencies (λ), cost (σ), conjugation frequency (γ) and θ for *E. coli* BW25113 carrying ancestral, evolved plasmid and variants using horizontal transfer (HT) model

| Strains | λ | | | σ | | | γ | | | θ^c^ | | | γ/θ^d^ | 1-((1-λ)/2^σ) | |
| --- | --- | --- | --- | --- | --- | --- | --- | --- | --- | --- | --- | --- | --- | --- | --- |
|  | CI^a^ Low  （2.5%） | MLE^b^ | CI High  (97.5%) | CI Low  (2.5%) | MLE | CI High  (97.5%) | CI Low  (2.5%) | MLE | CI High  (97.5%) | CI Low  (2.5%) | MLE | CI High  (97.5%) |  |  |  |
| BP_A_^e^ | 1.75E-07 | 9.05E-06 | 1.08E-04 | 5.00E-01 | 7.05E-01 | 1.22E+00 | 1.91E-03 | 8.59E-02 | 5.10E-01 | 2.74E-03 | 2.95E-01 | 1.61E+00 | 2.91E-01 | 3.87E-01 |  |
| BP_E_^f^ | 1.42E-06 | 1.72E-06 | 2.74E-05 | 9.56E-01 | 1.49E+00 | 3.63E+00 | 4.00E-01 | 7.32E-01 | 1.00E+00 | 7.42E-03 | 3.23E-01 | 6.84E-01 | 2.27E+00 | 6.44E-01 |  |

^a^ CI, confidence interval; ^b^ MLE, Maximum Likelihood Estimate; ^c^ θ, the fraction of the plasmid-carrying cells at which the frequency of conjugations is half its maximum; ^d^ γ/θ, transfer frequency threshold needed to guarantee the persistence of plasmids; ^e^ BP_A_, BW25113(pHNSHP24); ^f^ BP_E_, BW25113(pHNSHP24-36D).
