# Supplementary material for: The evolution of infectious transmission promotes the persistence of mcr-1 plasmids
Source: mBio. 2023 Jun 14;14(4):e00442-23. doi: 10.1128/mbio.00442-23 (PMC10470590; doi:10.1128/mbio.00442-23)
Supplement: Table S4 — Bacterial strains and plasmids used in this study. [file mbio.00442-23-s0006.docx]

**Table S4 Bacterial strains and plasmids used in this study**

| **Strains/plasmids** | **Genotype** | **References** |
| --- | --- | --- |
| *E. coli* C600(pHNSHP24) | *E. coli* C600 (*F^-^, thr, leu, fhuA, lacY, rpsL, supE*) harboring pHNSHP24 plasmid | (1) |
| *E. coli* BW25113 | $\Delta$(*araD-araB*)*567*, $\Delta$l*acZ4787*(::*rrnB-3*), λ^-^, *rph-1*,$\Delta$(*rhaD-rhaB*)*568*, *hsdR514* | (2) |
| *E. coli* BW25113::*kan* | A kanamycin-resistant gene was inserted in the truncated *lacZ* of *E. coli* BW25113 | This study |
| pHNSHP24 | A hybrid of an IncFII plasmid and a phage-like pO111 plasmid, colistin^R^ | This study |
| pHNSHP24$\Delta$*traJ* | pHNSHP24 with deletion of *traJ* | This study |
| pHNSHP24$\Delta$*cDmt* | pHNSHP24 with deletion of *cDmt* | This study |
| pHNSHP24-36D | Evolved pHNSHP24 plasmid with C1999T mutation in *cDmt* and A51G mutation in 5'UTR of *traJ* | This study |
| pHNSHP24-36D$\Delta$c*Dmt* | pHNSHP24-36D with deletion of *cDmt* | This study |
| pHNSHP24-36D$\Delta$c*Dmt*::*kan* | pHNSHP24-36D with deletion of *cDmt*, Kan^R^ | This study |
| pHNSHP24-14D | Evolved pHNSHP24 plasmid with C1999T mutation in *cDmt* | This study |
| pHNSHP24-7D | Evolved pHNSHP24 plasmid with C1999T mutation in *cDmt* | This study |
| pHNSHP24$\Delta$*mcr-1* | pHNSHP24 with deletion of *mcr-1* | This study |
| pHNSHP24-36D$\Delta$*mcr-1* | pHNSHP24-36D with deletion of *mcr-1* | This study |
| pHSG575 | *oriV*_psc101_ (Cm^R^) | (3) |
| pHSG575-*traJ* | pHSG575 carrying *traJ* with native promoter | This study |
| pHSG575-*traJ*(A51G) | pHSG575 carrying *traJ* with mutation A51G in 5'UTR | This study |
| pHGR01 | Containing promoterless *lacZ* (Kan^R^) | (4) |
| *P*_traJ_-lacZ | The upstream region (-217 to +242) of *traJ* was fused with promoterless *lacZ* | This study |
| *P*_traJ(A51G)_-lacZ | *P*_traJ_-lacZ with mutation (A-G) in +51 position of *traJ* | This study |
| *P*_traJ(A51C)_-lacZ | *P*_traJ_-lacZ with mutation (A-C) in +51 position of *traJ* | This study |
| *P*_traJ(C56U)_-lacZ | *P*_traJ_-lacZ with mutation (C-T) in +56 position of *traJ* | This study |
| pKD46 | *oriV*_psc101_(Ts); λRed recombinase expression vector | (2) |
| pCP20 | Flp recombinase expression vector (Cm^R^, Amp^R^) (Ts) | (2) |
| pKD4 | PCR template for λRed recombination system (Kan^R^) | (2) |

**References**

1. Wu R, Lv L, Wang C, Gao G, Yu K, Cai Z, Liu Y, Yang J, Liu JH. 2022.IS26-Mediated Formation of a Hybrid Plasmid Carrying *mcr-1.1*. Infect Drug Resist 15: 7227–7234.

2. Datsenko KA, Wanner BL. One-step inactivation of chromosomal genes in *Escherichia coli* K-12 using PCR products. *Proc Natl Acad Sci U S A*. 2000 Jun 6;97(12):6640-5.

3. Takeshita S, Sato M, Toba M, Masahashi W, Hashimoto-Gotoh T. High-copy-number and low-copy-number plasmid vectors for *lacZ* alpha-complementation and chloramphenicol- or kanamycin-resistance selection. *Gene*. 1987;61(1):63-74.

4. Fu H, Jin M, Ju L, Mao Y, Gao H. Evidence for function overlapping of CymA and the cytochrome bc1 complex in the *Shewanella oneidensis* nitrate and nitrite respiration. *Environ Microbiol*. 2014;16(10):3181-95.
