# Supplementary material for: The evolution of infectious transmission promotes the persistence of mcr-1 plasmids
Source: mBio. 2023 Jun 14;14(4):e00442-23. doi: 10.1128/mbio.00442-23 (PMC10470590; doi:10.1128/mbio.00442-23)
Supplement: Table S5 — Primers used in this study. [file mbio.00442-23-s0007.docx]

**Table S5 Primers used in this study**

| Primers | Sequence^a^ |  |
| --- | --- | --- |
| FrepB-F | TGATCGGTTTAAGGAATTTTG |  |
| FrepB-R | GAAGATCAGTCACACCATCC |  |
| *mcr-1*-F | TCGCGGCATTCGTTATA |  |
| *mcr-1*-R | GGTGGCGTTCAGCAGTC |  |
| pO111-F | TAAAATCTACCAATGTGGCGCTA |  |
| pO111-R | GCGCCCTAGTTTATCCAG |  |
| knock-traJ-F | TGGAACGATTTTTTCCAAAAAATGATGAGGAATAAAAAATGTAGGCTGGAGCTGCTTCG |  |
| knock-traJ-F | CATAATGTCACCTTGTTTTTAACAAAAACGAAACATCAAATGGGAATTAGCCATGGTCC |  |
| knock-cDmt-F | TGATGCTCTGGTAAATGCCCTTGGTGGAGAGGCTAAGCATGTAGGCTGGAGCTGCTTCG |  |
| knock-cDmt-R | ATCACGATTCATTGAGGCGATATTACACTAATAAATTTAATGGGAATTAGCCATGGTCC |  |
| insert-kan-F | TGATGTGCCCGGCTTCTGACCATGCGGTCGCGTTCGGTTGTGTAGGCTGGAGCTGCTTC |  |
| insert-kan-R | CTCCTGCACTGGATGGTGGCGCTGGATGGTAAGCCGCTGATGGGAATTAGCCATGGTCC |  |
| check-traJ-F | TTCTTGAACTGGGGCTTCGT |  |
| check-traJ-R | TAGCATCGTCCAATACCAGT |  |
| check-cDmt-F | AAAGTCGCTAACCATGACGAA |  |
| check-cDmt-R | TGGACGCAGATTTCTTCCTGT |  |
| check-kan-F | CTGGCGGTTAAATTGCCAAC |  |
| check-kan-R | TATCTGTTGTTTGTCGGTGAACG |  |
| pro-traJ-F | CCGGAATTCCGGATCAGTAGCGAAAATTTTGGG |  |
| pro-traJ-R | CCCAAGCTTGGGCTATTCAGCACATTTTAAAAT |  |
| lacZ-traJ-F | CCGGAATTCCGGTTGAGTCTCTAAGTCCCCATG |  |
| lacZ-traJ-R | CCCAAGCTTGCAGAGAATTTTTCATTGTAGCC |  |
| mu-24traJ(A51C)-R | TGCAGGGAGCGCGACCTCCCCGCATCGACTGTCCA | |
| mu-24traJ(A51C)-F | GGGAGGTCGCGCTCCCTGCATCGGCGATTTTTAAT | |
| mu-24traJ(C56U)-F | GGAGAGCGATCTCCCCGCATCGACTGTCCATAGAA |  |
| mu-24traJ(C56U)-R | ATGCGGGGAGATCGCTCTCCCTGCATCGGCGATTT |  |
| qtraY-F | CCTGCTCCGGGCAATAAAGT |  |
| qtraY-R | GTTTCCTCTGCTCCCTCCTTT |  |
| qtraA-F | ACGATACGGTTAAGGCGACC |  |
| qtraA-R | AACGACGCTCATACCCACAG |  |
| qtraC-F | GATGCCGTTAGTGGCGGATA |  |
| qtraC-R | ACCGCCATGTTGTAGTTGGT |  |
| qtraG-F | TGTCGCAACAGTTTGCACAG |  |
| qtraG-R | CCAGGCTGTACCTGTTCCTG |  |
| qtraD-F | ACAGCAAACTGGTGGACACA |  |
| qtraD-R | TGCAGGTAACGGATGGCTTT |  |
| qtraI-F | TGACGGAGTTCAGTCACAGC |  |
| qtraI-R | GTGTCCTGCCGGTATAGTCG |  |
| gapA-F | GTTGTCGCTGAAGCAACTGG |  |
| gapA-R | AGCGTTGGAAACGATGTCCT |  |

^a^Restriction sites are underlined.
